# Application of Artificial Intelligence in Shoulder Pathology

**DOI:** 10.3390/diagnostics14111091

**Published:** 2024-05-24

**Authors:** Cong Cheng, Xinzhi Liang, Dong Guo, Denghui Xie

**Affiliations:** 1 Department of Orthopaedics, People’s Hospital of Longhua, Shenzhen 518000, China; chengcong9090@163.com; 2 Department of Joint Surgery and Sports Medicine, Center for Orthopedic Surgery, Orthopedic Hospital of Guangdong Province, The Third Affiliated Hospital of Southern Medical University, Guangzhou 510630, China; kenliangxz@163.com (X.L.); 19927472465@163.com (D.G.); 3 Guangdong Provincial Key Laboratory of Bone and Joint Degeneration Diseases, The Third Affiliated Hospital of Southern Medical University, Guangzhou 510630, China

**Keywords:** shoulder pathology, artificial intelligence, modern medicine, clinical practice

## Abstract

Artificial intelligence (AI) refers to the science and engineering of creating intelligent machines for imitating and expanding human intelligence. Given the ongoing evolution of the multidisciplinary integration trend in modern medicine, numerous studies have investigated the power of AI to address orthopedic-specific problems. One particular area of investigation focuses on shoulder pathology, which is a range of disorders or abnormalities of the shoulder joint, causing pain, inflammation, stiffness, weakness, and reduced range of motion. There has not yet been a comprehensive review of the recent advancements in this field. Therefore, the purpose of this review is to evaluate current AI applications in shoulder pathology. This review mainly summarizes several crucial stages of the clinical practice, including predictive models and prognosis, diagnosis, treatment, and physical therapy. In addition, the challenges and future development of AI technology are also discussed.

## 1. Introduction

Artificial intelligence (AI) is the science and engineering of creating intelligent machines for imitating and expanding human intelligence. It is a branch of computer science that has the remarkable ability to perform tasks by simulating human cognitive functions [1]. Through the analysis and comparison of extensive datasets, AI technology has been engaged in innovative applications in the field of medicine and revolutionized approaches to various healthcare challenges [2,3,4].

In 1955, John McCarthy and his colleagues embarked on a research project to explore the feasibility of reproducing all aspects of human intelligence using a machine/computer with minimal human involvement [5]. This pivotal endeavor marked the establishment of the field of AI, which became the foundation of subsequent computer research and development. The early accomplishments of AI could be due to the fact that it was proficient in solving tasks that were easy to formally program but challenging for humans to execute [6,7]. Paradoxically, tasks that appeared effortless for humans showed greater challenges due to their reliance on intuitive processes, making them inherently arduous to formalize through coding [8,9].

**Figure 1:**
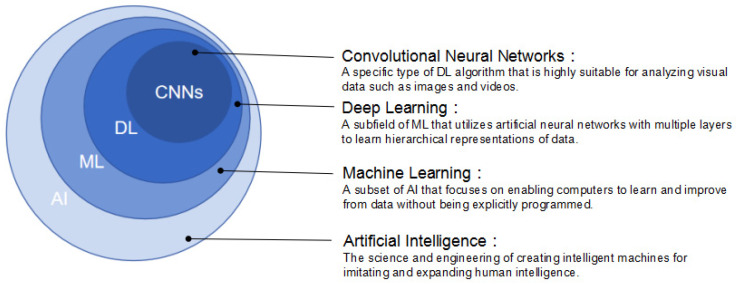
The relationship of AI, ML, DL, and CNNs.

Machine learning (ML) was first introduced as a subset of AI in 1959 (Figure 1) [10]. ML focuses on the development of algorithms and models that enable computers to learn and make decisions on the basis of input data. Instead of being explicitly programmed to perform a certain task, ML uses statistical techniques to learn from examples and adjust their internal parameters (weights) and progressively improves with experience [11]. According to the learning process, ML can be broadly classified into three groups: supervised learning, unsupervised learning, and reinforcement learning [12]. In supervised learning, the algorithm is trained on labeled data following explicit instructions, which means that it is given input–output pairs and learns to map the input to the output [13]. Unsupervised learning involves training on unlabeled data, and the algorithm should find patterns or structures within the data, which can potentially reveal hidden patterns yet to be recognized by humans [14]. In reinforcement learning, the algorithm was trained to make sequences of decisions by rewarding or punishing it based on its actions [15]. Additionally, some certain ML models lack interpretability or transparency, which is called the black box phenomenon. It means that although these models can make accurate predictions or classifications, the underlying reasoning or decision-making process is not easily understandable by humans [16]. Nevertheless, despite its complexities and occasional opacity, ML has immense impacts on various domains, ranging from image recognition and natural language processing to recommendation systems and autonomous vehicles [17].

Deep learning (DL), as a highly sophisticated advancement of ML, was proposed in the 1980s. It emerged from the neural network research conducted by Geoffrey Hinton and his colleagues [18]. It is a powerful methodology of unsupervised learning, and specifically designed to understand complex patterns and relationships in large datasets [19]. DL algorithms are inspired by the intricate connectivity and function of the human brain and are composed of artificial neural networks (ANNs) with multiple layers to perform complex tasks [20]. An ANN includes an input layer, multiple intermediate layers, and an output layer. Each layer comprises interconnected nodes, called artificial neurons or units, which process and transform the input data. The output of one layer acts as the input for the next layer, allowing the network to learn hierarchical representations of the data [21]. The key advantage of DL is its ability to automatically learn and extract relevant features from unstructured and unlabeled data and eliminate the need for manual feature engineering. By iteratively adjusting the weights and biases of the neural network during training, DL models can discover complex and abstract representations that capture intricate patterns present in the data [16,22]. The availability of large datasets and advances in computational power have contributed to the rapid growth and state-of-the-art performance in tasks like image classification, object detection, machine translation, and so on. [23,24]. 

Convolutional neural networks (CNNs) are a type of DL algorithm that is highly suitable for analyzing visual data such as images and videos [25]. CNNs are designed to automatically and adaptively learn spatial hierarchies of features from the input data through the use of convolutional layers, which apply filters (also known as kernels) to the input data to extract relevant features. These filters are learned during the training process, allowing the network to identify patterns such as edges, textures, and shapes at different scales [26,27]. CNNs also typically include other types of layers, such as pooling layers, which downsample the feature maps to reduce computational complexity, and fully connected layers [28]. 

**Figure 2:**
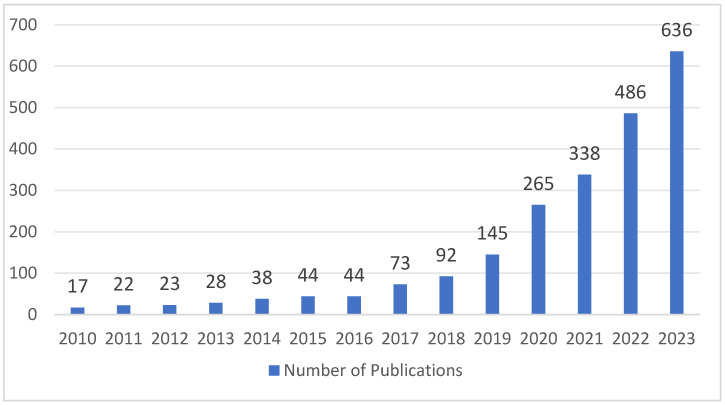
AI-related publications in the field of orthopedics from 2010 to 2023.

**Figure 3:**
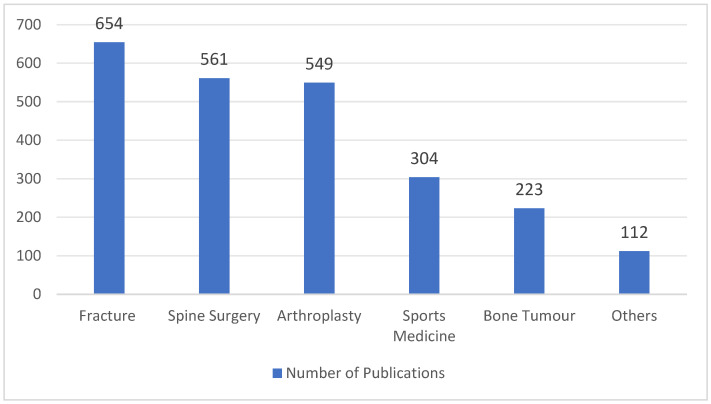
AI-related publications in the subspecialties of orthopedics from 2010 to 2023.

**Figure 4:**
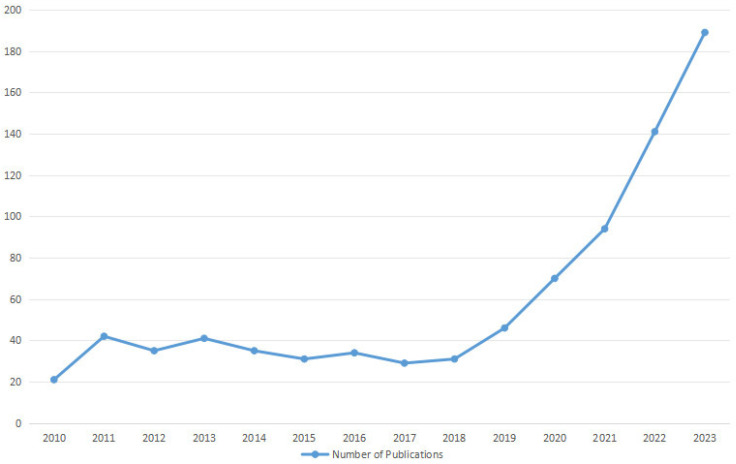
AI-related publications in the field of shoulder pathology from 2010 to 2023.

Over the last few years, AI technology has been integrated into the field of medicine for multiple purposes, such as clinical diagnosis, decision support, electronic health records, personalized treatments, drug discovery and development, patient care and assistance, and health monitoring [9,29]. It represents a significant growing trend poised to revolutionize various aspects of healthcare delivery, improve clinical outcomes, and transform the patient experience. The application of AI technology in the field of orthopedics has developed exponentially, with a ninefold increase in the number of publications between 2017 and 2021 (Figure 2). This can be attributed to advancements in computer science, processor speeds, and related technologies, which have facilitated the development of AI-driven tools applicable to orthopedics. Nowadays, AI technology has demonstrated remarkable utility in predictive analysis, medical imaging interpretation, preoperative planning, surgical planning, and postoperative care and rehabilitation in various subspecialties of orthopedics (Figure 3). One particular area of orthopedics focuses on shoulder pathology, which has experienced rapid growth, especially in the past five years [30] (Figure 4). Shoulder pathology refers to a range of disorders or abnormalities affecting the shoulder joint and surrounding soft tissues. These conditions can cause pain, inflammation, stiffness, weakness, and reduced range of motion. Some of the common shoulder pathologies include rotator cuff tears (RCTs), shoulder impingement syndrome (SIS), shoulder instability, shoulder osteoarthritis, and adhesive capsulitis [30,31,32].

Although the potential benefits of AI technology in the field of orthopedics are substantial, there are still several challenges and considerations that should be evaluated and addressed, such as data quality, interoperability, regulatory compliance, ethical considerations, and the need for interdisciplinary collaboration. To date, numerous studies have been conducted to review AI applications in some subspecialties of orthopedics [2,6,14,16,28,30]. However, there has not yet been a comprehensive review of the recent advancements in shoulder pathology. Therefore, the purpose of this review is to evaluate the current AI applications in the literature concerning shoulder pathologies. It will explore several crucial stages of the clinical process, including predictive models and prognosis, diagnosis, treatment, and physical therapy. In addition, the challenges and future development of AI technology are also discussed. A glossary of key terms associated with AI technology is provided in Table 1.

## 2. Methods

### 2.1. Search Strategy

A systematic literature review was conducted following the guidelines of the Preferred Reporting Items for Systematic Reviews and Meta-Analyses (PRISMA). One reviewer carried out structured searches on the PubMed, Google Scholar, and ScienceDirect databases to retrieve all relevant articles published from 1 January 2010 to 1 January 2024. The search query included the terms: (artificial intelligence OR machine learning OR deep learning) AND (shoulder OR shoulder pathology OR shoulder pain OR shoulder disorder OR shoulder surgery OR rotator cuff OR shoulder fracture OR shoulder tendinopathy). The titles, abstracts, and full-text articles were independently screened by two reviewers. The reference lists of the included articles were also reviewed and cross-referenced to identify any other additional relevant studies that were not retrieved through the keyword search. 

### 2.2. Eligibility Criteria and Article Selection

Study eligibility was determined using standardized inclusion and exclusion criteria. Disagreements or discrepancies were resolved through consensus. The inclusion criteria were as follows: (1) full-length original articles involving Al or ML or DL applications in shoulder pathologies; (2) diagnosis or treatment relevant to orthopedists; (3) randomized controlled trials (RCTs), non-randomized studies, or observational studies; (4) published in English. Exclusion criteria were as follows: (1) review articles, conference papers, book chapters, letters to the editor and (2) animal studies, post-mortem studies.

**Figure 5:**
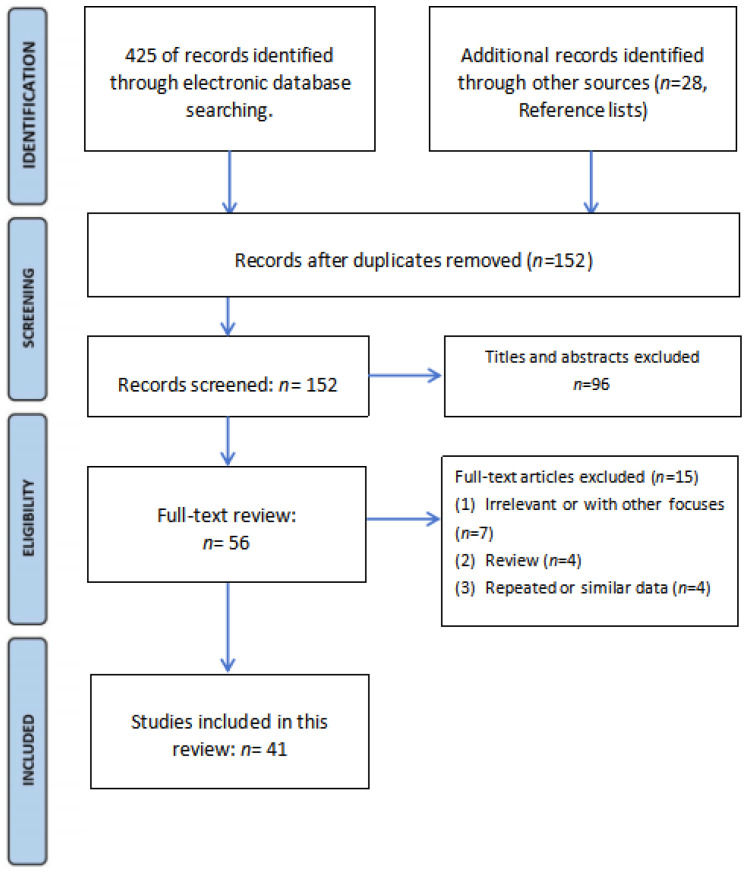
The flowchart for literature screening.

The screening process is shown in Figure 5. After the literature screening, 41 studies were included in this review.

## 3. Rotator Cuff Tears (RCTs)

### 3.1. Diagnosis

The diagnosis of RCTs is important for providing timely and accurate treatment for patients. AI technology could analyze medical images with high accuracy and reduce the risk of misdiagnosis. 

X-rays are widely recognized for their high negative predictive value (NPV) in diagnosing RCTs [33]. A specific DL algorithm was trained on a 6793 shoulder radiograph series and tested on a 1095 radiograph series by Kim et al. The results showed that the algorithm accurately ruled out significant RCTs based on shoulder radiographs, with a sensitivity of 97.3%, NPV of 96.6%, and a negative likelihood ratio (LR-) of 0.06. Subgroup analysis revealed that age < 60 years, non-dominant side, absence of trauma history, and ultrasound examination were associated with negative test results, and NPVs were higher in patients younger than 60 years and those examined with ultrasound [33]. In another study, a DL algorithm was developed to evaluate subscapularis tendon tears using axillary lateral shoulder radiography. A dataset of 2779 radiographs was used for training, and the algorithm outputted the probability of a subscapularis tendon tear exceeding 50% thickness. The algorithm’s performance was validated by two distinct test sets, with arthroscopy and MRI findings serving as the reference gold standard, respectively. Performance evaluation yielded an area under the curve (AUC) of 0.83 and 0.82 for two test sets. At the high-sensitivity cutoff point, the sensitivity was 91.4% and 90.2% and the NPV was 90.4% and 89.5% for the respective test sets. The algorithm successfully identified the subscapularis insertion site at the lesser tuberosity as the most sensitive region [34]. Iio et al. also developed a DL algorithm using shoulder radiography as a screening tool for RCTs, which showed high diagnostic performance for full-thickness tears, with an AUC of 0.82, sensitivity of 94.5%, NPV of 96.2%, and LR- of 0.10 [35]. 

Currently, magnetic resonance imaging (MRI) is widely recognized as the most efficient and reliable technique for examining RCTs without invasive procedures. DL has shown promise in accurately detecting and classifying RCTs on shoulder MRI scans. A DL model was developed using 11,925 MRI scans by Lin et al. [36]. The model achieved excellent performance, with an AUC of 0.93 for supraspinatus tears, 0.89 for infraspinatus tears, and 0.90 for subscapularis tears. Notably, it demonstrated high accuracy for full-thickness tears with AUCs of 0.98, 0.99, and 0.95 for the respective tendons.

Additionally, multisequence input yielded improved results for some tear types. The accuracy of the DL model compared favorably to specialized radiologists, highlighting its potential as a valuable tool in clinical practice.

DL is also a viable approach for the automated detection classification and segmentation of supraspinatus tears on MRI scans. A total of 200 shoulder MRI scans were retrospectively collected by Yao et al. [37], which contained full-thickness tears, partial-thickness tears, or intact supraspinatus tendons. The researchers developed a 3-stage pipeline, including a slice selection network, a segmentation network based on an encoder–decoder architecture (U-Net), and a custom multi-input classifier. The DL model achieved a sensitivity of 85.0%, specificity of 85.0%, AUC of 0.943, and dice similarity coefficient (DSC) of 0.814. No significant difference in accuracy was observed between 1.5 T and 3.0 T MRI scans.

CNNs play a crucial role in enhancing the analysis and interpretation of shoulder MRI scans. A 2D CNN model was developed by Guo et al. to automatically detect supraspinatus tears, trained on 701 shoulder MRIs and validated on 69 arthroplasty MRIs [38]. The model showed optimal performance, achieving high F1-scores and sensitivity on both surgery and internal test sets. Subgroup analyses confirmed its robustness across tear degrees and MRI field strengths. The comparison in diagnostic accuracy with clinicians revealed that the model was equivalent to senior clinicians and better than junior clinicians.

The 2D CNNs process data in two parameters, namely width and height, while 3D CNNs can capture more complex patterns and relationships in the data by incorporating the additional dimension (depth or time), making 3D CNNs more suitable for analyzing volumetric data. A 3D U-Net CNN model was developed to identify, segment, and visually represent RCTs in 3D, using MRI data from 303 patients with RCTs [39]. Two shoulder specialists labeled the RCTs in the entire MRIs using in-house developed software (Reconeasy 3D program, SeeAnn Solution, South Korea). The CNN model was trained following the augmentation of a training dataset and tested using randomly selected test data, maintaining a 6:2:2 ratio for training, validation, and test data. The 3D U-Net CNN successfully detected, segmented, and visualized RCT areas with a DSC of 94.3%, sensitivity of 97.1%, specificity of 95.0%, precision of 84.9%, F1-score of 90.5%, and Youden index of 91.8%. Thus, the proposed method demonstrated high accuracy and successful 3D visualization. Shim et al. [40] used the Voxception-ResNet (VRN) structure to train a 3D CNN model to automatically detect RCT presence, classify tear size, and visualize tear location in 3D on a dataset of MRI data from 2124 patients. The proposed method indicated the superiority over orthopedists in terms of accuracy and specificity. Moreover, the generated 3D class activation map (CAM) provides valuable information on tear localization and size.

Shoulder MRI using standard multiplanar sequences often requires a long scan time. However, accelerated sequences, although providing a shorter scan time, have limitations in terms of noise and resolution. To address this, DL-based reconstruction (DLR) has been proposed as a potential solution to reduce scan time while preserving image quality. In a retrospective study involving 105 patients who underwent 110 shoulder MRI examinations, standard sequences (scan time: 9 min 23 s) and accelerated sequences (scan time: 3 min 5 s; 67% reduction) were compared. The standard sequences were reconstructed conventionally, while the accelerated sequences were reconstructed using both conventional and DLR pipelines. Two radiologists evaluated the images for subjective image quality, artifacts, and specific pathologies. Diagnostic accuracy was assessed using arthroscopic findings as the reference standard in 27 patients who underwent arthroscopy. The results indicated that the accelerated sequences with DLR provided similar subjective image quality, artifacts, and diagnostic performance compared to standard sequences [41]. Liu et al. [42] showed a significantly reduced scan time (6 min 1 s vs. 11 min 25 s) and higher image quality in DLR MRI compared to the conventional method. The image quality satisfaction survey among 400 patients received high scores in DL-MRI from all radiologists. Kaniewska et al. [43] also declared that DLR could improve diagnostic accuracy and image quality with a thorough assessment of the subacromial bursa and good agreement for other shoulder structures. 

Ultrasound imaging has been identified as a valid alternative to MRI. Ultrasound imaging offers several advantages over MRI including real-time imaging, cost-effectiveness, wide availability, and dynamic assessment [44]. However, speckle noise can degrade image resolution in ultrasound imaging, making conventional vision-based algorithms ineffective for segmenting diseased regions. Lee et al. [45] proposed a novel fully CNN called Segmentation Model Adopting a pre-trained Classification Architecture (SMART-CA), which incorporated an integrated positive loss function (IPLF) to accurately diagnose the locations of RCTs using ultrasound imaging during orthopedic examinations. SMART-CA utilizes a pre-trained network to extract distinct features that improve segmentation accuracy. IPLF efficiently optimizes SMART-CA for imbalanced datasets like RCT. The experimental results indicated that SMART-CA with IPLF achieved improved precision, recall, and DSC, and is robust for segmentation in the presence of speckle noise, outperforming the existing state-of-the-art networks. In another study, a total of 194 ultrasound images were used to train and test five pre-trained CNN models. Among them, DenseNet121 demonstrated the best classification performance with 88.2% accuracy, 93.8% sensitivity, 83.6% specificity, and an AUC score of 0.832. A gradient-weighted class activation mapping (Grad-CAM) highlighted the sensitive features in the learning process on ultrasound images [46]. 

### 3.2. Predictive Models and Prognosis

In addition to the diagnosis of RCTs, AI technology has been applied in the predictive models to evaluate the functional and anatomical outcomes according to various pre-operative factors. The occupation ratio and fatty infiltration of the supraspinatus muscle are crucial parameters for predicting the diagnosis and treatment prognosis of RCTs. Ro et al. [47] employed a DL model to segment the supraspinatus muscle and fossa regions by quantitatively measuring the occupation ratio of the supraspinatus muscle and calculating the amount of fatty infiltration of the supraspinatus muscle using the Otsu thresholding technique on MRI scans. The model exhibited high DSC, accuracy, sensitivity, and specificity in the segmentation. The fatty infiltration measure significantly varied across different Goutallier grades [48]. Furthermore, a strong negative correlation was observed between occupation ratio and fatty infiltration. Kim et al. [49] also proposed a DL model from an MRI dataset of 240 patients with various disease severities to detect the supraspinatus muscle and fossa regions, which achieved high accuracy and DSC. They declared that this model could assist clinicians to accurately track the preoperative and postoperative changes in muscle volume of the supraspinatus fossa. Besides evaluating an MRI dataset, Taghizadeh et al. [50] developed and verified a CNN model that can automatically measure and characterize the degeneration of rotator cuff muscles in a total of 103 shoulder CT scans from 95 patients with glenohumeral osteoarthritis. The automatic CNN segmentation showed comparable DSC to the manual ones. The CNN model also quantified muscle atrophy, fatty infiltration, and overall muscle degeneration rapidly, providing accurate and reliable predictions. In addition, Medina et al. [51] performed automated segmentations of shoulder MRI images using two CNN models, in which Model A was created for Y-view selection, and Model B was for muscle segmentation. They concluded that the combination of deep CNN models could achieve overall accurate and reliable Y-view selection and automated algorithm muscle segmentation.

In the context of identifying the relationship between important clinical features and the prediction of RCTs, Li et al. [52] conducted a retrospective trial including patients with shoulder pain and dysfunction who underwent questionnaires and physical examinations in outpatient settings. Six ML models were developed and assessed using accuracy, AUC, and Brier scores. Among them, the XGBoost model exhibited superior performance. Moreover, the Shapley plot highlighted the Jobe test, bear hug test, and age as the most important variables in predicting RCTs. Potty et al. [53] created an ML model to predict post-operative functional outcomes following arthroscopic rotator cuff repair by collecting pre-operative and post-operative patient data. The proposed model successfully predicted post-operative scores accurately. The most essential features in predicting patient recovery were identified as pre-operative American Shoulder and Elbow Surgeons (ASES) score, pre-operative pain score, body mass index (BMI), age, and tendon quality. They declared that it is valuable for pre-operative counseling, planning, and resource allocation. The main studies of AI applications in the RCTs are summarized in Table 2.

### 3.3. Physical Therapy

Physical therapy has been established as an effective treatment for RCTs, resulting in significant improvements in patient-reported outcomes and reducing the need for surgery. However, poor adherence to physical therapy programs became a challenge to effectively managing common shoulder disorders, particularly with unsupervised home exercise programs [30]. To address this, twenty healthy adults without prior shoulder disorders participated in the study of Burns et al. [54], performing seven exercises from an evidence-based rotator cuff physiotherapy protocol while data from a 6-axis inertial sensor on the active extremity were collected. Four supervised DL algorithms were trained and optimized within an activity recognition chain framework to classify the exercises. The algorithms’ performance was evaluated using 5-fold cross-validation, first temporally and then by subject. All algorithms achieved a categorical classification accuracy of over 94% in the temporally stratified cross-validation, with the convolutional recurrent neural network (CRNN) algorithm performing the best at 99.4%. They proved the technical feasibility of using such an approach to monitor and assess adherence to shoulder physiotherapy exercise protocols at home.

## 4. Shoulder Instability

### 4.1. Diagnosis

To date, there is no definitive evidence regarding glenohumeral translation in dynamic glenohumeral joint stability models. Therefore, a standardized method for assessing shoulder kinematics can provide a clear understanding and be beneficial for patient treatment. Croci et al. [55] obtained fluoroscopic images for both shoulders of 12 participants with unilateral RCTs and 13 patients who were asymptomatic subjects. They designed a 3D full-resolution CNN (nnU-Net) model to automatically locate five landmarks (glenohumeral joint center, humeral shaft, inferior and superior edges of the glenoid, calibration sphere, and the most lateral point of the acromion). As a result, the model achieved accurate landmark detection, with all landmarks and the calibration sphere located within 1.5 mm, except for the humeral landmark with a difference of 9.6 mm. This proposed model provides a reliable and efficient means of automatically identifying and tracking anatomical landmarks, enabling the measurement of clinically relevant anatomical configurations and investigation of dynamic glenohumeral joint stability in pathological shoulders. 

In terms of assessing osseous injuries associated with anterior shoulder instability, CT scans of the shoulder with 3D reconstruction are considered the gold standard. The CT scans provide improved conceptualization and accurate quantification of injuries at the glenoid and humeral head [56]. However, this method exposes patients to much radiation. An alternative approach involving the use of 3D MRI models has been advocated recently, which can be obtained and reconstructed during standard 2D MRI of the shoulder. The 3D MRI models have demonstrated equal effectiveness in evaluating bipolar bone loss [57]. Rodrigues et al. [58] collected shoulder MRI images from 100 patients and developed a fully automated segmentation 3D CNN model for proton density-weighted images. The CNN model showed high accuracy in segmenting the humerus and glenoid, and in the evaluation of glenohumeral anatomy and GBL.

Wei et al. [59] collected radiographs of 106 elbows and 140 shoulders, half of which had dislocations. Multiple CNN models were trained and tested using datasets from external hospitals and online radiology repositories. The CNN models achieved high accuracy in identifying joint dislocations, with AUCs greater than 0.99 on internal test sets and greater than 0.97 on external test sets. The CAMs indicated that the CNNs accurately identified relevant joints regardless of the presence of dislocations with excellent generalizability to external test sets.

### 4.2. Predictive Models and Prognosis

The predictors of optimized functional outcomes after surgery for anterior shoulder instability from a global perspective, rather than domain-specific perspectives, remain elusive. Till et al. [60] used ML clustering to identify predictors for achieving the “optimal observed outcome” after surgery for anterior shoulder instability. Medical records, images, and operative data of patients under 40 years old were analyzed. Of the 200 patients with an average follow-up of 11 years, 64% achieved the “optimal observed outcome” characterized by reduced postoperative pain, low rates of recurrent instability, revision surgery, osteoarthritis, and improved range of motion. Additionally, 41% achieved a “perfect outcome” across all categories. Negative predictors included a longer time from initial instability to presentation and habitual/voluntary instability, while a predilection toward preoperative subluxations was a positive predictor. 

## 5. Rotator Cuff Calcific Tendinopathy (RCCT)

RCCT is one of the most common causes of shoulder pain. It is characterized by the deposition of calcium hydroxyapatite crystals either inside or around rotator cuff tendons. Although published studies have highlighted a wide range of risk factors for the onset of RCCT, including endocrine disorders, hyperlipidemia, and sports strain, the etiology of symptomatic RCCT is currently debatable [61,62].

Ultrasound imaging is regarded as an excellent imaging tool to visualize calcifications within the rotator cuff tendons. Vassalou et al. [63] evaluated the performance of two ML models in predicting long-term complete pain resolution following ultrasound-guided percutaneous irrigation of calcific tendinopathy (US-PICT) in 100 patients with rotator cuff disease. The two models incorporated data related to procedural details, patient characteristics, and calcification properties to predict pain at 1 year post-US-PICT. The results showed an AUC of 69.2% for predicting complete pain resolution at 1 year, with age and baseline VAS scores being the most influential variables. Furthermore, the inclusion of VAS scores at 1 month did not significantly improve the models’ performance, indicating that the models could be beneficial in predicting patient outcomes following US-PICT. Chiu et al. [64] declared that their DL model was able to assist clinicians in diagnosing supraspinatus calcific tendinopathy during ultrasound examinations with high accuracy, sensitivity, and specificity.

## 6. Proximal Humeral Fractures (PHFs)

Accurate diagnosis and classification of PHFs are essential for appropriate treatment planning. The complexity arises from the variability in fracture patterns, which can make it difficult to precisely determine the fracture type based solely on visual inspection. Factors such as overlapping bone fragments, subtle displacement, and the presence of associated injuries can further complicate the classification process [63,64]. 

Automation of fracture classification with AI technology has been proven to improve diagnostic accuracy, reduce inter-observer variability, and accelerate the classification process. A CNN model trained by Chung et al. [65] demonstrated exceptional performance, with a top-1 accuracy of 96% and an AUC of 1.00 for distinguishing PHFs from normal shoulder radiographs. Additionally, the CNN model showed promising results in classifying fracture types based on the Neer classification system, achieving top-1 accuracy ranging from 65% to 86% and AUC ranging from 0.90 to 0.98. Furthermore, the model outperformed orthopedists in both detection and classification tasks. Notably, the CNN model’s superiority was more obvious in complex three-part and four-part fractures. Magneli et al. [66] also evaluate the classification performance of the CNN model for PHFs based on the AO/OTA classification system. The overall AUC for fracture classification was 0.89, including excellent AUC for diaphyseal humerus fractures (0.97), clavicle fractures (0.96), and good AUC for scapula fractures (0.87), which showed that the proposed model could effectively utilize plain radiographs and classify fractures. Dipnall et al. [67] assessed the classification performance of several ML algorithms based on the Neer classification system from six input text datasets, including X-ray and/or CT scan data and patient age and/or sex information. They declared that these ML algorithms achieved satisfactory performance, with one special model exhibiting good accuracy at 61% and an excellent one-versus-rest score above 0.8, providing valuable assistance to radiologists and orthopedists by speeding up the classification process.

## 7. Other Shoulder Pathologies

Scapulohumeral periarthritis, also known as periarthritis of the shoulder, is characterized by a gradual development of shoulder pain, which is more pronounced at night, with limited functions [68]. Yu et al. [69] examined the efficiency of combining an intelligent clustering analysis algorithm with musculoskeletal ultrasound imaging for the differential diagnosis and rehabilitation of scapulohumeral periarthritis. The thickness and clarity of the shoulder posterior capsule were observed in different pain groups. Factors such as musculoskeletal ultrasound parameters, length of service, work nature, and work busyness significantly influenced shoulder periarthritis pain. The proposed intelligent algorithm indicated promising accuracy, sensitivity, and specificity when tested on clinical ultrasound samples. 

Subacromial impingement syndrome (SIS) is another common disorder causing shoulder pain. Shu et al. [70] included 17 participants performing shoulder abduction and adduction while their ultrasound images were captured. The CNN model accurately depicted the trajectory of the humeral greater tubercle in relation to the lateral acromion. Subacromial motion metrics from dynamic ultrasonography were extracted using different CNN models. Consequently, the self-transfer learning-based (STL) CNN model performed better than the traditional CNN model. The errors in measuring the minimal vertical acromiohumeral distance were significantly smaller using the STL-CNN models. This study successfully demonstrated the feasibility of using the CNN model for automatic detection of anatomical landmarks and capturing essential motion metrics in dynamic shoulder ultrasonography, which was helpful in diagnosing SIS. Jiang et al. [71] included 10 radiomic features for radiomics model construction in the ML-based ultrasomic analysis of SIS stage evaluation. They stated that the ML-derived ultrasomics model could provide reliable stage evaluations in patients with SIS. 

Shoulder pain attributed to inflammation of the long head of the biceps tendon is a prevalent condition. Bicipital peritendinous effusion (BPE) is the most frequently occurring abnormality associated with the biceps tendon and is connected to different shoulder injuries [72]. Obtaining a clear and accurate ultrasound image is difficult for inexperienced radiologists [73]. An automated BPE recognition system was designed by Lin et al. [74] to classify inflammation into four categories: normal, mild, moderate, and severe in ultrasound imaging. Three experiments were accordingly conducted to validate the classification performance of the recognition system under different settings and situations. Ultimately, the proposed CNN model achieved an accuracy of 75% for three-class BPE classification (normal, moderate, and severe) and revealed comparable results to other state-of-the-art methods.

Grauhan et al. [75] trained a CNN model to detect the most common causes of acute or chronic shoulder pain in 2700 plain radiographs, which were reviewed and labeled for six findings. The developed CNN model achieved high accuracy, with an AUC of 0.871 for PHFs, 0.896 for joint dislocation, 0.945 for osteoarthritis, and 0.800 for periarticular calcifications. It also demonstrated near-perfect accuracy in detecting osteosynthesis and endoprosthesis, with AUC 0.998 and 1.0, respectively, supporting that such a CNN model could provide additional assistance and safety for the clinicians on duty.

## 8. Future of AI

AI technology is a high-tech production that adapts to the development of the contemporary era, and it represents a significant milestone in the ongoing scientific and technical revolution. Similar to the steam and electrical revolutions of the past, AI is transforming human life and driving societal progress.

The future development of AI technology in shoulder diagnosis and treatment is promising. AI technology will continue to evolve and improve in its ability to analyze medical imaging, which enables faster and more accurate identification of degenerative diseases, fractures, and other shoulder pathologies. It also will facilitate personalized preoperative planning by analyzing patient data, including medical imaging, clinical records, and biomechanical parameters. Clinicians can utilize AI-based simulation tools to optimize surgical plans, predict outcomes, and simulate procedures virtually. Moreover, AI-powered surgical robots will become increasingly sophisticated, assisting orthopedic surgeons with precise intraoperative navigation, implant placement, and tissue manipulation, enhancing surgical accuracy, reducing complications, and enabling minimally invasive procedures, ultimately improving patient recovery times and postoperative outcomes. In addition, AI-powered wearable devices and sensors can track shoulder function, range of motion, and strength during rehabilitation. These data can be analyzed by AI models to provide personalized feedback, exercise recommendations, and progress tracking. It can also help monitor patients remotely, allowing for early identification of any complications or deviations from the expected recovery trajectory [76].

However, despite the rapid advancement and potential benefits of AI technology, its widespread application has been hindered by several factors. The main concerns are the need for retraining, associated costs, and a perceived lack of comprehensive education, contributing to a reluctance of clinicians to engage with the technological tools available fully, thus influencing their integration into clinical practice [77]. Thus, providing comprehensive education and training programs tailored to clinicians can help alleviate concerns and foster greater confidence in utilizing AI technology. 

In addition, AI models often deal with vast amounts of sensitive patient data. Ensuring the security of these data is paramount to prevent unauthorized access, breaches, or misuse that could compromise patient confidentiality and trust in the healthcare system. Legislative measures should be taken to establish guidelines for the collection, storage, and usage of the data. Many AI algorithms, particularly DL models, operate as black boxes, making it difficult to interpret their decisions and predictions [78]. Additionally, AI algorithms should be robust and reliable across diverse patient populations and clinical settings. Thus, it is difficult to keep their reliability and generalizability. In the face of resistance to adopting new technologies or changes in workflow processes, integrating AI solutions into existing clinical workflows shows logistical and organizational challenges. Implementing AI technology can be costly and resource-intensive, particularly for smaller healthcare organizations with limited budgets and infrastructure. The regulatory and legal frameworks are complex and evolving. Compliance with regulatory requirements, such as data privacy and security and medical device regulations, adds another challenge to AI deployment for developers and healthcare organizations [16,79]. 

Ethical issues should be taken into consideration in the application of AI technology to ensure patient well-being, safety, privacy, and fairness [80]. All patients regardless of socioeconomic background have equitable access to AI-powered healthcare solutions. Telehealth and remote monitoring technologies are able to offer promising avenues for extending healthcare services to remote or marginalized communities, bridging geographical divides and improving healthcare access. Continual monitoring and evaluation should be conducted to assess their impact on patient care, identify potential biases or errors, and make necessary improvements to enhance ethical performance. AI technology should prioritize patient safety and the delivery of high-quality care by rigorous testing, validation, and monitoring to minimize errors and improve patient outcomes. It should also be transparent and explainable in its development, validation, and deployment, including disclosing potential limitations, biases, and uncertainties associated with AI predictions or recommendations, respecting patients’ autonomy and rights to make informed decisions. 

There are several potential limitations in the current data available for training AI models for shoulder pathology. Firstly, the quality of medical imaging data can vary significantly due to image artifacts, positioning errors, and inconsistent acquisition protocols, potentially compromising the efficacy of AI models trained on such data. Furthermore, access to large and diverse datasets is often limited, leading to potential issues of overfitting and inadequate generalization. Manual annotation of medical images for labeling shoulder pathologies is both time-consuming and prone to subjectivity, resulting in potential errors or inconsistencies that may affect the reliability of AI model predictions. Moreover, the datasets may exhibit biases towards certain patient demographics, imaging modalities, or healthcare institutions, which can significantly impact the AI model’s performance and exacerbate healthcare disparities. Longitudinal data tracking the progression of shoulder pathologies over time is valuable for understanding disease trajectories and predicting treatment outcomes. However, such data may be scarce or fragmented, making it difficult to train AI models to predict disease progression accurately.

## Figures and Tables

**Table 1:** Glossary of key terms.

Term	Definition
Area under the curve (AUC)	A valuable metric for evaluating the performance of binary classification models, which provides a concise measure of the model’s ability to discriminate between positive and negative classes and is widely used for comparing and assessing the overall performance of predictive models.
Class activation map (CAM)	A technique that generates a heatmap to visualize the important regions of an input image for predicting a specific class in a deep convolutional neural network, and helps in interpreting model decisions and understanding the features learned by the network during the classification process.
DenseNet121	DenseNet is a deep learning architecture characterized by dense connectivity patterns, where each layer receives direct input from all preceding layers, leading to improved feature reuse, parameter efficiency, and gradient flow during training. DenseNet121 has 121 layers in total and is commonly used for image classification tasks on the ImageNet dataset.
Dice similarity coefficient (DSC)	A statistical measure used to quantify the similarity between two sets, often employed in the context of image segmentation to evaluate the overlap between predicted and ground truth masks, ranging from 0 to 1, with 1 indicating perfect overlap between the two sets and 0 indicating no overlap.
F1-score	A performance metric used to evaluate the accuracy of binary classification models, which is to predict one of two possible outcomes based on input data, with values ranging from 0 to 1, where 1 indicates perfect precision and recall and 0 indicates poor performance.
Gradient-weighted class activation mapping (Grad-CAM)	A technique that extends the class activation map (CAM) approach to provide better visual explanations for the predictions made by deep convolutional neural networks.
nnU-Net	An extension of the original U-Net architecture and a framework for 3D biomedical image segmentation that aims to provide a standardized and automated way to train and evaluate deep learning models on various datasets.
Otsu thresholding technique	An image processing technique used for automatic image thresholding, and the goal of thresholding is to separate objects or regions of interest from the background in an image by converting it into a binary image (black and white).
Segmentation Model Adopting a pre-trained Classification Architecture (SMART-CA)	A deep learning algorithm that improves the efficiency and accuracy of CNNs by adaptively refining the network architecture during training based on the complexity of the input data, which uses a self-modulating mechanism and a measure of network capacity called the channel attention score to achieve this.
Shapley plot	A valuable tool for explaining and interpreting machine learning models by attributing the model’s predictions to individual features, help data scientists and stakeholders gain insights into the model’s decision-making process, and understand the significance of each feature in driving the model’s output.
U-Net	A convolutional neural network architecture that was designed for biomedical image segmentation tasks. The U-Net architecture consists of a contracting path to capture context and a symmetric expanding path to enable precise localization.
Voxception-ResNet (VRN)	A hybrid neural network architecture that merges the strengths of Voxception and ResNet to tackle tasks that require processing 3D image data.
XGBoost model	A versatile and efficient algorithm that excels in handling structured/tabular data and is widely used for tasks such as regression, classification, ranking, and more.
Youden index	A single statistic that captures the performance of a binary classification test, which takes into account both the sensitivity and specificity of the test to provide an overall measure of its accuracy, with 1 indicating perfect performance and 0 indicating no discriminatory power.

**Table 2:** AI applications in the rotator cuff tears.

Author (Year)	Input Feature	Model/Algorithm	Dataset	Type of Outcome	Results
Kim et al., 2020 [33]	X-ray	ResNet-based CNN ^(1)^	6793 radiograph series	Rule out significant RCTs ^(2)^	The sensitivity, NPV ^(3)^, and LR- ^(4)^ were 97.3%, 96.6%, and 0.06, respectively.
Kang et al., 2021 [34]	X-ray	ResNet-based CNN	2779 radiograph series	Rule out subscapularis tendon tears	The AUC ^(4)^, sensitivity, NPV, and LR- were 0.83 91.4%, 90.4%, and 0.21 in Test Set 1, and 0.82 90.2%, 89.5%, and 0.21 in Test Set 2, respectively.
Iio et al., 2023 [35]	X-ray	EfficientNet-based CNN	2803 radiograph series	Rule out significant RCTs	The sensitivity, NPV, and LR- were 94.5%, 96.2%, and 0.10, respectively.
Lin et al., 2023 [36]	MRI	ResNet-based CNN	11,925 MRI scans	Detection and classification of RCTs	The AUCs for supraspinatus, infraspinatus, and subscapularis tendon tears were 0.93, 0.89, and 0.90, respectively. The model performed best for full-thickness supraspinatus, infraspinatus, and subscapularis tears with AUCs of 0.98, 0.99, and 0.95, respectively.
Yao et al., 2022 [37]	MRI	ResNet-based CNN	200 MRI scans	Detection and segmentation of supraspinatus tears	The sensitivity and specificity were 85.0% and 85.0%, respectively. The AUC for classification was 0.943; DSC ^(5)^ for segmentation was 0.814.
Guo et al., 2023 [38]	MRI	Xception-based CNN	701 MRI scans for training and 69 MRI scans for clinical validation	Detection of supraspinatus tears	The model showed high F1-scores and sensitivity on both surgery and internal test sets. Subgroup analyses confirmed its robustness across tear degrees and MRI field strengths.
Lee et al., 2020 [39]	MRI	U-Net-based CNN	303 MRI scans	Segmentation of RCTs	The model reached 94.3% DSC, 97.1% sensitivity, 95.0% specificity, 84.9% precision, 90.5% F1-score, and a Youden index of 91.8%.
Shim et al., 2020 [40]	MRI	VRN ^(6)^-based CNN	2124 MRI scans	Detect the presence or absence of RCTs, classify the tear size, and provide 3D visualization of the tear location.	The model outperformed orthopedists in binary accuracy (92.5% vs. 76.4% and 68.2%), top-1 accuracy (69.0% vs. 45.8% and 30.5%), top-1 ± 1 accuracy (87.5% vs. 79.8% and 71.0%), sensitivity (0.94 vs. 0.86 and 0.90), and specificity (0.90 vs. 0.58 and 0.29). The generated 3D CAM ^(7)^ provided effective information regarding the 3D location and size of the tear.
Lee et al., 2021 [45]	Ultrasound imaging	VGG19-basedCNN, denoted as SMART-CA ^(8)^	1400 ultrasound images	Segmentation of RCTs	The precision, recall, and DSC were 0.604% (+38.4%), 0.942% (+14.0%), and 0.736% (+38.6%), respectively.
Ho et al., 2022 [46]	Ultrasound imaging	CNN (based on VGG19, ResNet50, InceptionV3, DenseNet121, or Xception)	194 ultrasound images	Segmentation of RCTs	DenseNet121 demonstrated the best performance, with 88.2% accuracy, 93.8% sensitivity, 83.6% specificity, and an AUC score of 0.832.
Ro et al., 2021 [47]	MRI	VGG19-based CNN	240 MRI scans	Segmentation of the supraspinatus muscle and fossa, and calculation of the amount of fatty infiltration of the supraspinatus muscle	The mean DSC, accuracy, sensitivity, specificity, and relative area difference for the segmented lesion were 0.97, 99.84, 96.89, 99.92, and 0.07, respectively, for the supraspinatus fossa and 0.94, 99.89, 93.34, 99.95, and 2.03, respectively, for the supraspinatus muscle.
Kim et al., 2019 [49]	MRI	CNN (fully convolutional network)	240 MRI scans	Segmentation of the supraspinatus muscle and fossa	The DSC is 0.9718 ± 0.012 in the fossa region and 0.9463 ± 0.047 in the muscle region.
Taghizadeh et al., 2020 [50]	CT	U-Net-based CNN	103 CT scans	Segmentation of RC ^(9)^ muscle and calculation of muscle atrophy and degeneration.	Average DSC for muscle segmentations (88 ± 9%) and manually by human raters (89 ± 6%) were comparable. The model provided good–very good estimates of muscle atrophy (R^2^ = 0.87), fatty infiltration (R^2^ = 0.91), and overall muscle degeneration (R^2^ = 0.91)
Medina et al., 2020 [51]	MRI	U-Net-based CNN	258 cases of model A (Y-view selection) and 1048 sagittal T1 Y-views for model (muscle segmentation)	Segmentation of RC muscles on a Y-view	Model A showed top-3 accuracy >98% to select an appropriate Y-view. Model B produced accurate RC muscle segmentations with mean DSC > 0.93.
Li et al., 2023 [52]	Questionnaires and physical examinations	ML (stacking, gradient boosting machine, bagging, random forest, XGBoost, and adaptive boosting)	1684 patients	Identify best model and important clinical variables for predicting patients with RCTs in outpatient settings.	The XGBoost model showed superior performance, with accuracy, AUC, and Brier scores of 0.85, 0.92, and 0.15, respectively. The most important variables were Jobe test, bear hug test, and age for prediction, with mean SHAP ^(10)^ values of 1.458, 0.950, and 0.790, respectively.
Potty et al., 2023 [53]	Patient-related and surgical-related factors	ML (linear regression, ridge regression, lasso, support vector regression, K-nearest neighbor, random forest, and XGBoost)	631 patients	Identify important clinical variables for predicting patients with repairing RCTs.	The XGBoost model predicted post-operative outcomes accurately. The most essential variables were pre-operative ASES ^(11)^ score, pre-operative pain score, BMI ^(12)^, age, and tendon quality.

^(1)^ Convolutional neural network, ^(2)^ rotator cuff tears, ^(3)^ negative predictive value, ^(4)^ area under the curve, ^(5)^ dice similarity coefficient, ^(6)^ Voxception-ResNet, rotator cuff, ^(7)^ class activation map, ^(8)^ Segmentation Model Adopting a pre-trained Classification Architecture, ^(9)^ rotator cuff, ^(10)^ Shapley additive explanation, ^(11)^ American Shoulder and Elbow Surgeons, ^(12)^ body mass index.

## Data Availability

Data are contained within the article.
